# SPDEF Inhibits Prostate Carcinogenesis by Disrupting a Positive Feedback Loop in Regulation of the Foxm1 Oncogene

**DOI:** 10.1371/journal.pgen.1004656

**Published:** 2014-09-25

**Authors:** Xin-Hua Cheng, Markaisa Black, Vladimir Ustiyan, Tien Le, Logan Fulford, Anusha Sridharan, Mario Medvedovic, Vladimir V. Kalinichenko, Jeffrey A. Whitsett, Tanya V. Kalin

**Affiliations:** 1 Division of Pulmonary Biology, the Perinatal Institute of Cincinnati Children's Research Foundation, Cincinnati, Ohio, United States of America; 2 Department of Environmental Health, College of Medicine, University of Cincinnati, Cincinnati, Ohio, United States of America; University of Washington, United States of America

## Abstract

SAM-pointed domain-containing ETS transcription factor (SPDEF) is expressed in normal prostate epithelium. While its expression changes during prostate carcinogenesis (PCa), the role of SPDEF in prostate cancer remains controversial due to the lack of genetic mouse models. In present study, we generated transgenic mice with the loss- or gain-of-function of SPDEF in prostate epithelium to demonstrate that SPDEF functions as tumor suppressor in prostate cancer. Loss of SPDEF increased cancer progression and tumor cell proliferation, whereas over-expression of SPDEF in prostate epithelium inhibited carcinogenesis and reduced tumor cell proliferation *in vivo* and *in vitro*. Transgenic over-expression of SPDEF inhibited mRNA and protein levels of Foxm1, a transcription factor critical for tumor cell proliferation, and reduced expression of Foxm1 target genes, including *Cdc25b*, *Cyclin B1*, *Cyclin A2, Plk-1, AuroraB, CKS1* and *Topo2alpha*. Deletion of SPDEF in transgenic mice and cultures prostate tumor cells increased expression of Foxm1 and its target genes. Furthermore, an inverse correlation between SPDEF and Foxm1 levels was found in human prostate cancers. The two-gene signature of low *SPDEF* and high *FoxM1* predicted poor survival in prostate cancer patients. Mechanistically, SPDEF bound to, and inhibited transcriptional activity of *Foxm1* promoter by interfering with the ability of Foxm1 to activate its own promoter through auto-regulatory site located in the −745/−660 bp *Foxm1* promoter region. Re-expression of Foxm1 restored cellular proliferation in the SPDEF-positive cancer cells and rescued progression of SPDEF-positive tumors in mouse prostates. Altogether, SPDEF inhibits prostate carcinogenesis by preventing Foxm1-regulated proliferation of prostate tumor cells. The present study identified novel crosstalk between SPDEF tumor suppressor and Foxm1 oncogene and demonstrated that this crosstalk is required for tumor cell proliferation during progression of prostate cancer *in vivo*.

## Author Summary

Development of prostate cancer is a multistep process that involves the loss of tumor suppressor functions and activation of oncogenes. SPDEF transcription factor is expressed in normal prostate epithelium and its expression changes during prostate carcinogenesis (PCa). Since the role of SPDEF in PCa remains controversial, we generated transgenic mice with loss- and gain-of-function of SPDEF to demonstrate that SPDEF functions as a tumor suppressor in PCa. In animal models, the loss of SPDEF promoted PCa and increased the levels of Foxm1, a well-known oncogenic protein. Overexpression of SPDEF in prostate epithelium decreased PCa and reduced Foxm1 levels. Proliferation defects in SPDEF-containing tumor cells were corrected by re-expression of Foxm1, providing direct evidence that SPDEF inhibits tumor cell proliferation through Foxm1. We further showed that SPDEF directly bound to Foxm1 promoter and prevented its auto-regulatory activation. In prostate cancer patients, the low SPDEF and high Foxm1 were found in most aggressive prostate tumors that were associated with poor prognosis. The combined two-gene signature of low SPDEF and high Foxm1 was a strong predictor of survival in prostate cancer patients. The present study identified novel molecular mechanism of prostate cancer progression, providing a crosstalk between SPDEF tumor suppressor and Foxm1 oncogene.

## Introduction

Development of cancer is a multistep process that involves gain-of-function mutations in oncogenes and inactivation of tumor suppressor genes, leading to increased tumor cell proliferation, survival and resistance to cell cycle arrest [1]. In normal prostate epithelium, relatively low rates of cell proliferation are balanced by a low rate of apoptosis [2]. In contrast, prostatic intraepithelial neoplasia (PIN) and early invasive carcinomas are characterized by an increase in the proliferation rate. Advanced and/or metastatic prostate cancers also display a significant decrease in the rate of apoptosis. Altered cell-cycle control plays a key role in progression of prostate cancer. Published studies have demonstrated significant activation of the PI3K/Akt and Erk mitogen-activated protein kinase (MAPK) signaling pathways in prostate carcinomas [3], [4] and the loss of PTEN tumor suppressor [5].

The transgenic adenocarcinoma of the mouse prostate (TRAMP) model recapitulates multiple stages of human PCa by using the *probasin* promoter to drive the expression of the *SV40* virus large and small T antigen (Tag) oncoprotein in prostate epithelial cells [6]. Tag inactivates the tumor suppressor proteins retinoblastoma (Rb), p53, and PP2A serine/threonine–specific phosphatase [7], inducing prostate tumors in adult mice. *SV40* T antigens also induce expression of the Foxm1 oncogenic protein, a member of the Forkhead Box (Fox) family of transcription factors [8]. Foxm1 is activated by the Ras/Erk signaling pathway [9] and transcriptionally induces cell cycle-regulatory genes, including *Cdc25b*, *Cyclin B1*, *Plk1*, *Aurora B*. Recent studies demonstrated that Foxm1 is required for initiation and progression of various cancers, including prostate cancer [9]–[11].

SPDEF (SAM pointed domain containing ETS transcription factor) belongs to the family of ETS transcription factors containing a conserved DNA binding domain (ETS domain). The ETS domain binds to a conserved central “GGA” trinucleotide motif [12]. SPDEF expression is restricted to the epithelial layers of the prostate or other lumen-containing organs including lung, breast, ovary, stomach and colon [13]–[16]. SPDEF regulates mucus secretion, goblet cell differentiation, tumor progression and metastasis [13], [16]–[20]. In prostate, SPDEF directly interacts with the androgen receptor functioning as a co-activator to induce prostate-specific antigen (PSA) in LNCaP prostate tumor cells [21]. The prostate-specific Nkx3.1 nuclear protein directly inhibits SPDEF and prevents SPDEF-mediated PSA activation, indicating a potential role of SPDEF in prostate cancer [22].

Currently, the role of SPDEF in cancer pathogenesis remains controversial. Both, reduced expression of SPDEF in prostate, breast, ovarian and colon tumors [23]–[25], and increased SPDEF in breast, ovarian and prostate tumors [15], [25]–[27] has been reported. Expression of SPDEF *in vitro* in either PC3 prostate or MDA-MB231 breast carcinoma cells decreased cellular proliferation and increased apoptosis [24], [28]. On the other hand, transfection of MCF10A and MCF12A breast carcinoma cells with SPDEF increased cell growth, cell invasiveness and tumorigenicity [26]. It is unclear whether SPDEF functions as tumor suppressor or oncogene in prostate carcinogenesis. Given the apparent lack of *in vivo* data using animal models, we generated several distinct mouse models of prostate cancer with loss-of-function and gain-of-function of SPDEF to demonstrate that SPDEF inhibits prostate carcinogenesis by preventing a positive feedback mechanism regulating the Foxm1 oncogene.

## Results

### Prostate carcinogenesis is increased in SPDEF^−/−^ mice

**Figure 1:**
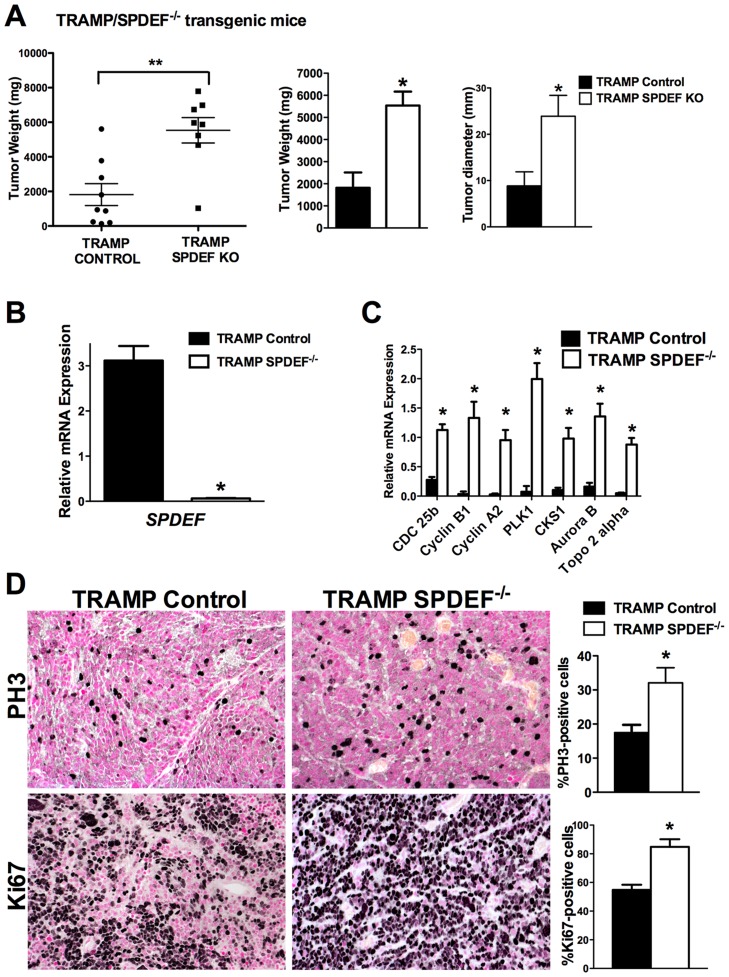
Prostate carcinogenesis is increased in SPDEF^−/−^ mice. Experimental *TRAMP/SPDEF^−/−^* and control *TRAMP* mice were sacrificed at 23 weeks of age. **A**. Deletion of *SPDEF* increased the weight and sizes of prostate glands. Mean weights and diameters of prostate glands (±SD) were calculated from 8–9 mouse prostates per group. **B**. Efficiency of *SPDEF* deletion is shown by qRT-PCR. Total prostate RNA was prepared from *TRAMP/SPDEF^−/−^* and *TRAMP* mice. β-actin mRNA was used for normalization. Data represent means ± SD of three independent determinations using prostate tissue from n = 5–10 mice in each group. **C**. Increased mRNA levels of *Cdc25b*, *Cyclin B1, Cyclin A2*, *Plk-1, CKS1, Aurora B* and *Topo2-alpha* were found in *TRAMP/SPDEF^−/−^* prostates by qRT-PCR. Data represent means ± SD of three independent determinations (n = 5–10 mice in each group). **D**. Increased cellular proliferation in *TRAMP/SPDEF^−/−^* prostates. Mouse prostate glands were harvested 23 weeks after birth and used for immunohistochemistry with Ki-67 and PH3 antibodies. Number of positive cells were counted in 5 random microscope fields (n = 6 mice per group). Data represent mean ± SD. A *p* value<0.01 is shown with (**) and *p* value<0.05 is shown with (*). Magnification: panels D, 200×.

To determine the role of SPDEF during prostate carcinogenesis *in vivo*, we crossed *SPDEF^−/−^* mice [14] with transgenic *TRAMP* mice that express SV40 T large and small antigens under the control of probasin promoter to drive oncogenic transformation of prostate epithelial cells [7]. *SPDEF^−/−^/TRAMP* and control *TRAMP* mouse prostates were analyzed at 23 weeks of age. A significant increase in prostate weight and size was observed in *SPDEF^−/−^/TRAMP* mice compared to *TRAMP* mice (Figure 1A). *SPDEF* mRNA was undetectable in *SPDEF^−/−^/TRAMP* prostates, confirming the efficient knockout of *SPDEF* (Figure 1B). The numbers of PH3-positive and Ki67-positive cells were increased in *SPDEF^−/−^/TRAMP* tumors, indicating the increased cellular proliferation in *SPDEF^−/−^/TRAMP* prostates (Figure 1D). Moreover, the mRNA levels of several proliferation-specific genes, such as *Cdc25b*, *Cyclin B1*, *Cyclin A2*, *Plk1*, *Cks1*, *Aurora B* and *Topo 2 alpha*, were increased in the *SPDEF^−/−^/TRAMP* prostates, a finding consistent with the increased cellular proliferation (Figure 1C). In the absence of TRAMP transgene, *SPDEF^−/−^* mice did not develop prostate tumors or PINs, and expression of proliferation-specific genes in *SPDEF^−/−^* prostates was unchanged (Figure S1A–B). Thus, the loss of SPDEF was not sufficient to initiate prostate tumors; however SPDEF deficiency promoted prostate carcinogenesis in the TRAMP mouse model. These results suggest that SPDEF functions as tumor suppressor in SV40 T-antigen induced prostate cancer.

### Expression of SPDEF in prostate adenocarcinoma cells decreased carcinogenesis in an orthotopic model

**Figure 2:**
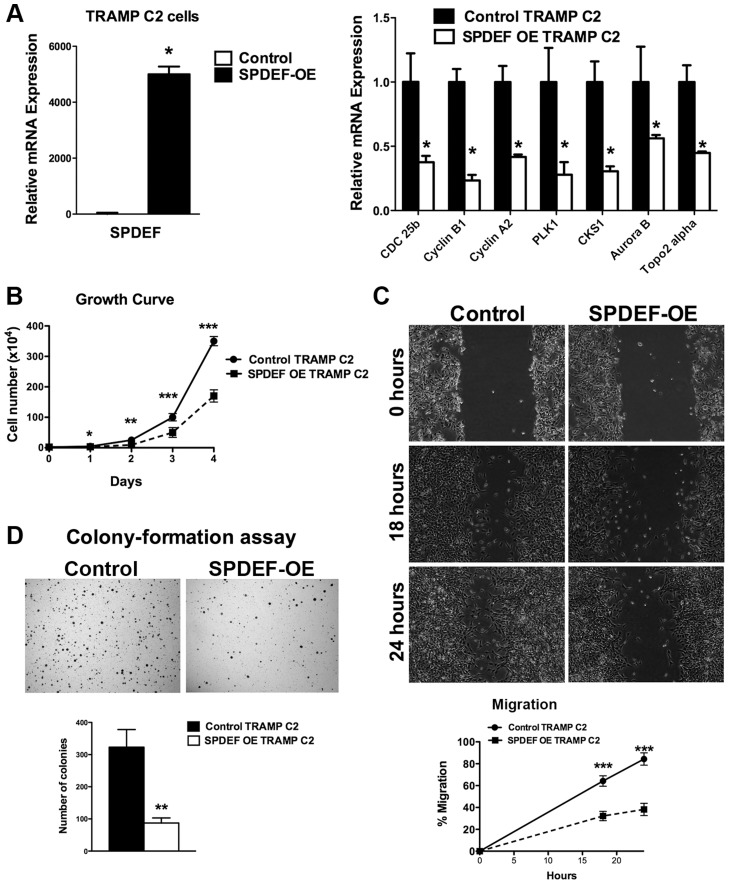
Expression of SPDEF in TRAMP C2 prostate adenocarcinoma cells decreased cell growth, reduced migration and colony formation on soft agar. **A**. qRT-PCR shows that *SPDEF* mRNA is increased in SPDEF OE cells (left panel). Overexpression of SPDEF reduced mRNAs of cell cycle regulatory genes. *β-actin* mRNA was used for normalization. **B**. Overexpression of SPDEF decreased proliferation of TRAMP C2 adenocarcinoma cells *in vitro*. Control and SPDEF-expressed TRAMP C2 cells were seeded in triplicates and counted at different time points using hemocytometer. **C**. Overexpression of SPDEF decreased migration of TRAMP C2 adenocarcinoma cells *in vitro*. Wound healing assay was used to measure cell migration. **D**. Increased expression of SPDEF decreased colony formation of TRAMP C2 cells on soft agar. The number of colonies were counted in 5 random fields in each of 3 individual wells per group. Data represent mean ± SD of three independent experiments. A *p* value<0.01 is shown with (**) and *p* value<0.05 is shown with (*).

We next determined whether the transgenic expression of SPDEF was sufficient to inhibit prostate carcinogenesis. Since TRAMP C2 prostate adenocarcinoma cells do not express endogenous SPDEF, we used SPDEF lentivirus to generate TRAMP C2 cells with the stable over-expression of SPDEF (SPDEF OE, Figure 2A). Over-expression of SPDEF decreased cellular proliferation and decreased mRNA encoding several proliferation-specific genes in cultured TRAMP C2 cells (Figure 2A–B). Moreover, expression of SPDEF decreased cell migration and reduced anchorage-independent growth of TRAMP C2 cells on soft agar (Figure 2C–D). Similar effects were observed in MycCap prostate adenocarcinoma cells (Figure S2). Thus, SPDEF may function as a tumor suppressor in prostate adenocarcinoma cells.

**Figure 3:**
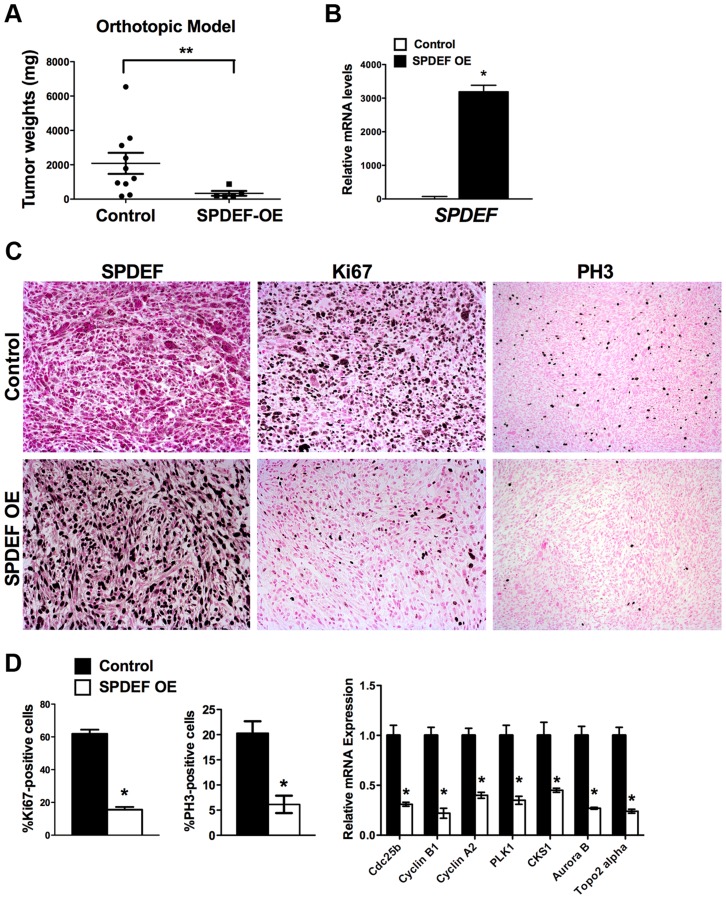
Expression of SPDEF in prostate adenocarcinoma cells decreased prostate carcinogenesis in orthotopic model. Mouse prostates were harvested 5 weeks after inoculation of control TRAMP C2 adenocarcinoma cells or TRAMP C2 cells with stable expression of SPDEF (SPDEF OE). **A**. SPDEF decreased the growth of prostate tumors in orthotopic model. Mean weights of prostate glands (±SD) are shown (n = 10 for control TRAMP C2 cells, n = 5 for SPDEF OE cells). **B**. mRNA levels of *SPDEF* in tumors are shown by qRT-PCR. **C**. SPDEF decreased cellular proliferation as demonstrated by reduced numbers of Ki-67-positive and PH3-positive cells. Magnification is ×100. **D**. Percentage of Ki-67-positive and PH3-positive cells were counted in five random microscope fields (n = 3 mice per group, left panels). Decreased mRNA levels of proliferation-specific genes in SPDEF OE prostates were found by qRT-PCR (right panel). Data represent means ± SD of three independent determinations using prostate tissue from n = 5–10 mice in each group. A *p* value<0.01 is shown with (**) and *p* value<0.05 is shown with (*).

To determine whether expression of SPDEF is sufficient to inhibit prostate carcinogenesis *in vivo*, SPDEF-overexpressing TRAMP C2 cells (SPDEF OE) were injected into prostates of syngeneic C57BL/6 mice and their tumorigenic potential was compared to parental TRAMP C2 cells. In this orthotopic model, high levels of SPDEF mRNA and protein were maintained in SPDEF OE tumors (Figure 3B and Figure 3C, left panels). Expression of SPDEF reduced the tumor burden (Figure 3A), decreased the number of Ki67 and PH3-positive cells (Figure 3C and 3D, left panels), and reduced mRNA levels of proliferation-specific genes *Cdc25b*, *Cyclin B1*, *Cyclin A2*, *PLK1*, *CKS1*, *Aurora B* and *Topo2 alpha* in the prostate tumors (Figure 3D, right panel). Altogether, our data indicate that overexpression of SPDEF decreased proliferation of TRAMP C2 and MycCap cancer cells and inhibited prostate carcinogenesis in the orthotopic model.

### Transgenic expression of SPDEF in prostate epithelium decreased prostate carcinogenesis

**Figure 4:**
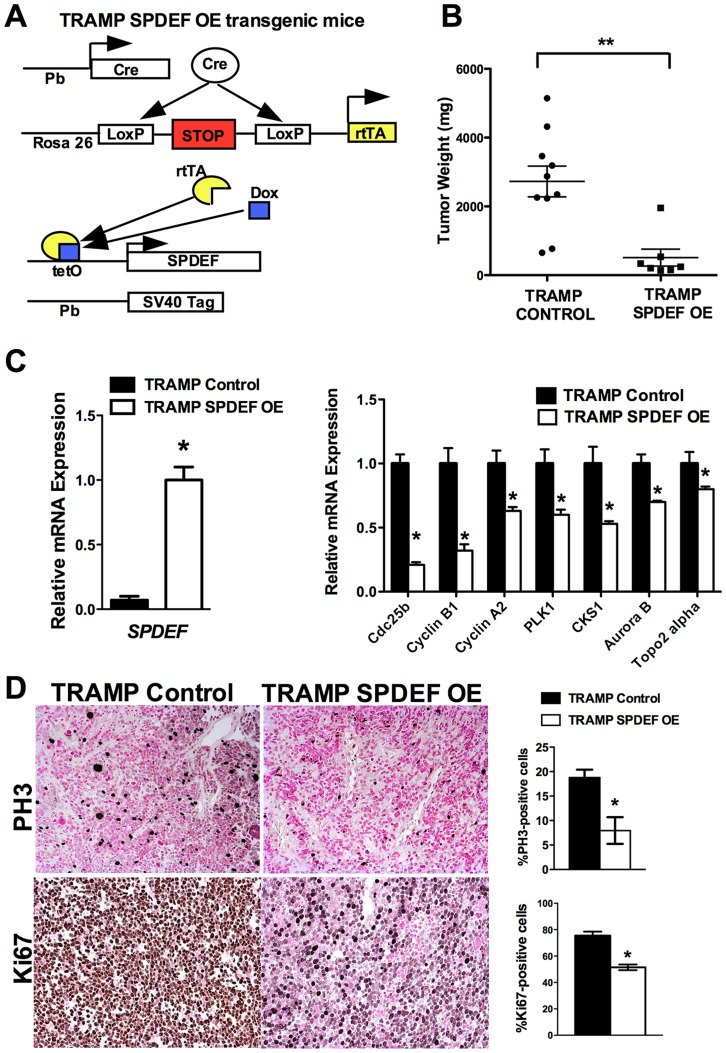
Transgenic expression of SPDEF in prostate epithelium decreased prostate carcinogenesis. **A**. Schematic drawing shows Dox-inducible expression of SPDEF and TRAMP transgenes in prostate epithelial cells. **B**. Decreased prostate carcinogenesis in TRAMP/SPDEF OE mice. Experimental *TRAMP/SPDEF OE* and control *TRAMP* mice were given Dox at 4 weeks of age and sacrificed at 25 weeks of age. Mean weight of prostate glands (±SD) was calculated from 7–10 mouse prostates per group. A *p* value<0.01 is shown with asterisk (**). **C**. TRAMP/SPDEF OE prostates show increased SPDEF mRNA (left panel) and decreased mRNA levels of cell cycle regulatory genes (right panel). **D**. Decreased number of proliferating cells in TRAMP/SPDEF OE prostates. Prostate sections were stained with PH3 antibody. The number of PH3-positive cells was counted using 5 random fields in each of 3 individual mice per group. Data represent mean ± SD. A *p* value<0.01 is shown with (**) and *p* value<0.05 is shown with (*). Magnification: panels D, 200×.

We generated transgenic mice with prostate epithelial-specific expression of SPDEF under Doxycycline (Dox) control. These transgenic mice contained *TRE-SPDEF* [13], *LoxP-stop-LoxP-rtTA(Rosa26)* and the *Probasin-Cre* transgenes (*Pb-Cre^tg/+^/LoxP-stop-LoxP-rtTA(Rosa26)^tg/tg^*/*TRE-SPDEF* mice, abbreviated as *SPDEF OE*). In *SPDEF OE* mice, Dox treatment induced the expression of SPDEF in prostate epithelial cells through excision of the LoxP-stop-LoxP cassette by the Probasin-driven Cre recombinase (Figure 4A). *SPDEF OE* mice were healthy and fertile, their prostates were normal. To induce prostate cancer, the *SPDEF OE* mice were bred with *TRAMP* transgenic mice to generate *TRAMP/SPDEF OE* mice (Figure 4A). SPDEF mRNA was increased in *TRAMP/SPDEF OE* mice after Dox treatment (Figure 4C). Expression of SPDEF in TRAMP mice was sufficient to inhibit prostate carcinogenesis as demonstrated by decreased tumor weight in *TRAMP/SPDEF OE* mice when compared to *TRAMP* mice (Figure 4B). The number of PH3-positive cells in *TRAMP/SPDEF OE* prostate tumors was reduced by 60% (Figure 4D). Moreover, the mRNA of several cell cycle regulatory genes, such as *Cdc25b*, *Cyclin B1*, *Cyclin A2*, *Plk1*, *Cks1*, *Aurora B*, and *Topo 2 alpha* were decreased in prostates of *TRAMP/SPDEF OE* mice (Figure 4C, right panel), a finding consistent with decreased cellular proliferation and decreased tumor sizes. These results indicate that increased expression of SPDEF in prostate epithelium is sufficient to decrease prostate carcinogenesis in the TRAMP mouse model.

### SPDEF inhibits Foxm1 expression during prostate carcinogenesis

**Figure 5:**
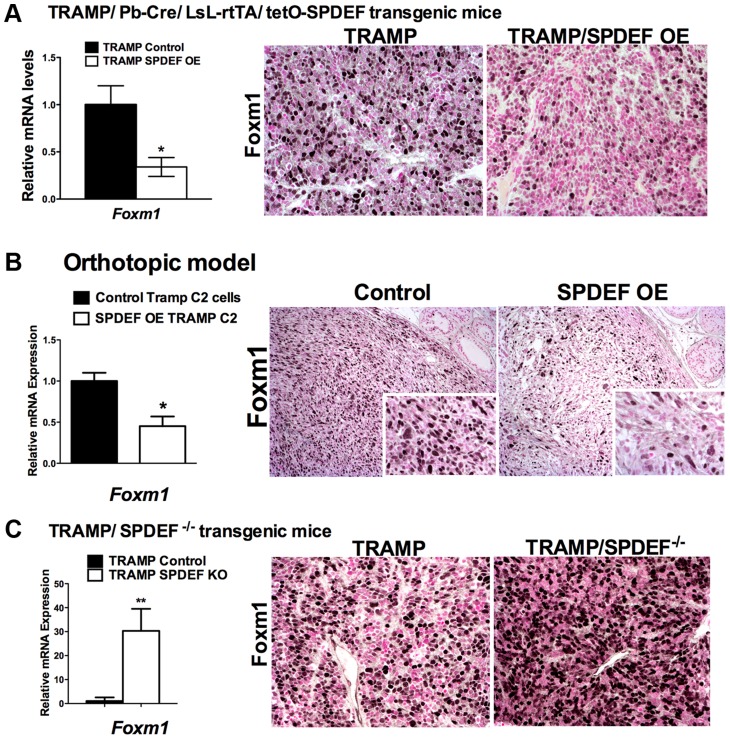
SPDEF and Foxm1 are inversely correlated in prostate carcinogenesis. **A**. In transgenic *TRAMP/SPDEF OE* mice, over-expression of SPDEF in prostate epithelial cells decreased Foxm1 mRNA (left panel) and protein (right panels) in prostate tumors. Experimental *TRAMP/SPDEF OE* and control *TRAMP* mice were sacrificed at 25 weeks of age. **B**. In orthotopic mouse model, SPDEF inhibited Foxm1 mRNA and protein levels during prostate carcinogenesis. Lentiviral expression of SPDEF in TRAMP C2 prostate adenocarcinoma cells decreased Foxm1 mRNA shown by qRT-PCR. *β-actin* mRNA was used for normalization. The decrease of Foxm1 staining in prostate tumors is shown by immunohistochemistry. Mouse prostates were harvested 5 weeks after inoculation of either control TRAMP C2 cells or TRAMP C2 cells expressing SPDEF (SPDEF OE). **C**. In transgenic *TRAMP/SPDEF^−/−^* mice, depletion of SPDEF increased Foxm1 mRNA (left panel) and protein levels (right panels). Experimental *TRAMP/SPDEF^−/−^* and control *TRAMP* mice were sacrificed at 23 weeks of age. Data represent means ± SD of three independent determinations (n = 3–5 mice in each group). Magnification: 200×. A *p* value<0.01 is shown with (**) and *p* value<0.05 is shown with (*). **C**. Magnification: 200×. A *p* value<0.01 is shown with (*).

**Figure 6:**
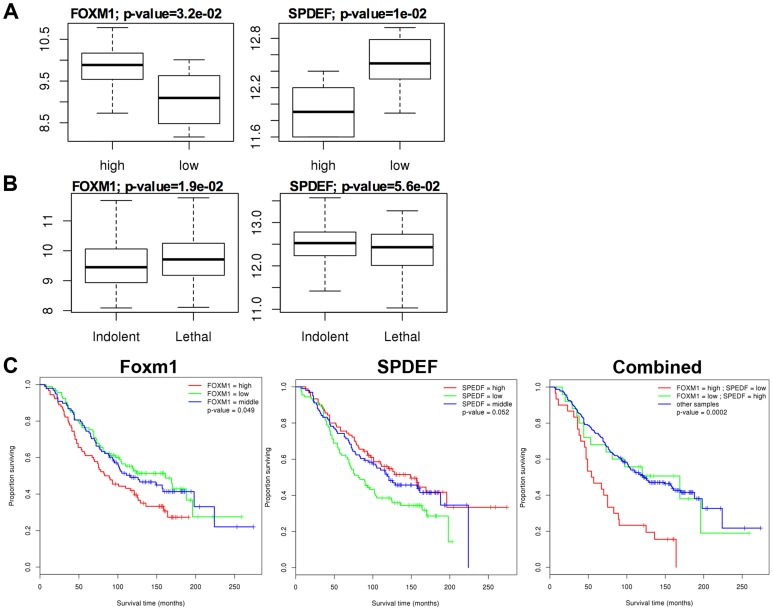
*SPDEF* expression is inversely correlated with *Foxm1* expression in human prostate cancer and of prognostic value for the prostate carcinoma patient survival. The data from two human prostate cancer microarray datasets, GSE21034 [30] and GSE16560 [31] were downloaded from the GEO archive. Expression levels were compared between Indolent and Lethal prostate cancers (**A**), and High-risk and Low-risk sample groups (**B**). High-risk samples were derived from patients with surviving less than 12 months and low-risk samples from patients surviving more than 192 months. **C**. Two-gene expression signature predicts poor patient survival. Kaplan-Meier survival analysis of prostate cancer patients using dataset GSE16560 [31]. Patients were stratified by the expression level of FOXM1 or SPEDF, or both together. The group with “high” FOXM1 and “low” SPDEF expression had the worst outcome (median survival time of 55.5 months).

Our *in vitro* and *in vivo* studies demonstrated that SPDEF inhibits *Cdc25b*, *Cyclin B1*, *Cyclin A2*, *Plk1*, *Cks1*, *Aurora B*, and Topo 2 alpha mRNAs, all of which are known targets of Foxm1 transcription factor [29]. Since Foxm1 is up-regulated in mouse and human prostate cancers and is required for prostate carcinogenesis [8], [10], we tested whether SPDEF inhibits Foxm1. In transgenic *TRAMP/SPDEF OE* mice, overexpression of SPDEF in prostate decreased Foxm1 mRNA and protein (Figure 5A). Consistent with findings in the transgenic mice, expression of SPDEF in TRAMP C2 cells decreased Foxm1 mRNA and protein expression in orthotopic prostate tumors (Figure 5B). Deletion of SPDEF in TRAMP/SPDEF^−/−^ mouse prostates caused a 30-fold increase in *Foxm1* mRNA and increased Foxm1 staining (Figure 5C). Finally, an inverse correlation between SPDEF and Foxm1 was found in human prostate cancers using two independent human prostate cancer microarray datasets, GSE21034 [30] and GSE16560 [31]. Expression levels of SPDEF and Foxm1 were compared between indolent and lethal prostate cancers and high-risk and low-risk sample groups (Figure 6A–B). High-risk samples were derived from patients with surviving less than 12 months and low-risk samples from patients surviving more than 192 months [32]. In high-risk group of patients and in lethal prostate cancers, Foxm1 mRNA was significantly overexpressed while SPDEF mRNA was significantly decreased (Figure 6A). Inverse correlation between Foxm1 and SPDEF expression levels was also found when metastatic and primary tumor samples were compared (Figure S3). The Kaplan-Meier survival analysis demonstrated that, on its own, the low SPDEF expression or high Foxm1 expression are associated with worse overall survival (Figure 6C). However, combined two-gene signature of low SPDEF and high Foxm1 is a much stronger predictor of survival (Figure 6C). These results suggest the existence of inverse correlation between SPDEF and Foxm1 expression in mouse and human prostate cancers.

### Re-expression of Foxm1 in the SPDEF-positive prostate adenocarcinoma cells restored tumor cell proliferation

**Figure 7:**
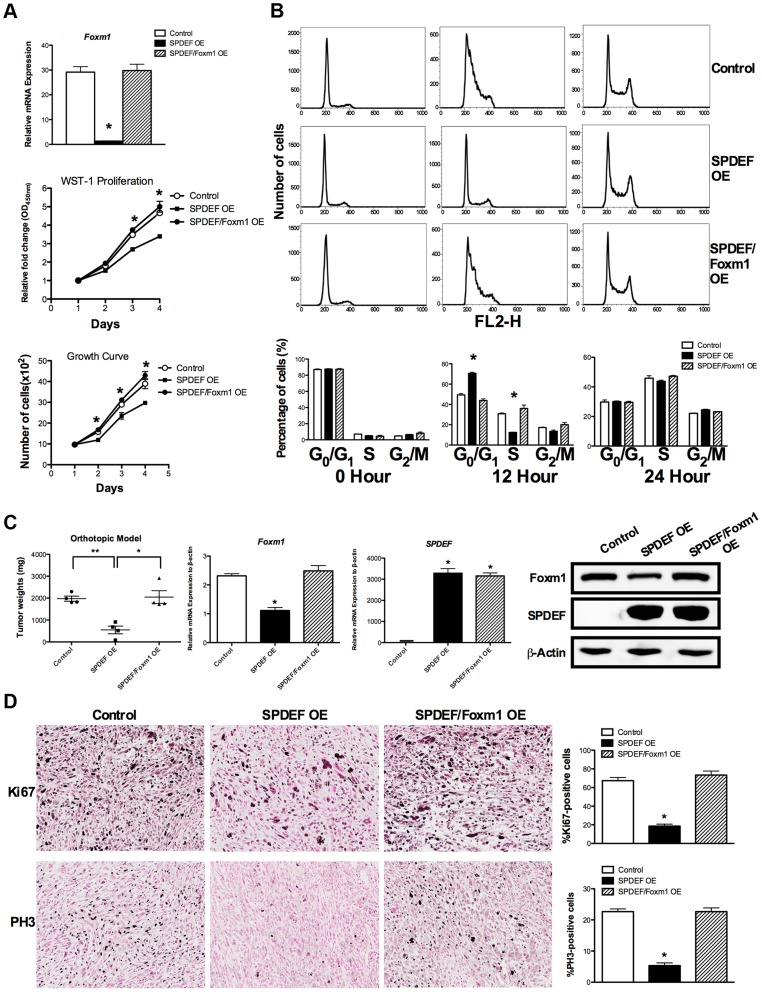
Re-expression of Foxm1 in the SPDEF-positive prostate adenocarcinoma cells restored tumor cell proliferation *in vitro* and *in vivo*. We used SPDEF-overexpressing prostate adenocarcinoma TRAMP C2 cells (SPDEF OE cells) to stably express Foxm1 (SPDEF/Foxm1 OE cells). **A**. Foxm1 mRNA levels were determined by qRT-PCR. Growth curves demonstrated that re-expression of Foxm1 in SPDEF OE cells restored the growth of these cells in culture. **B**. Flow cytometery shows that the re-expression of Foxm1 in SPDEF OE cells increased entry of synchronized cells into S phase at 12 hours after serum addition *in vitro*. **C**. In orthotopic mouse model of prostate cancer, re-expression of Foxm1 in SPDEF-overexpressing cancer cells restored tumor sizes that have been decreased after SPDEF expression (left panel). Foxm1 and SPDEF mRNAs in tumor tissues are shown by qRT-PCR (middle panels). Protein levels of Foxm1 and SPDEF are shown by Western blot (right panel). **D**. Re-expression of Foxm1 restored cellular proliferation in SPDEF-overexpressing prostate tumor cells as demonstrated by increased numbers of Ki-67-positive (upper panels) and PH3-positive (bottom panels) cells. Percentages of Ki-67-positive (upper panels) and PH3-positive cells (bottom panels) were counted in five random microscope fields (n = 3 mice per group, right panels). Magnification is ×100. A *p* value<0.01 is shown with (**) and *p* value<0.05 is shown with (*).

We next examined Foxm1 levels in SPDEF OE TRAMP C2 cells *in vitro*. A 30-fold reduction in *Foxm1* mRNA was observed in SPDEF OE cells compared to control cells expressing empty vector (Figure 7A, upper panel). Decreased Foxm1 levels in the SPDEF OE cells were associated with reduced cell proliferation (Figure 7A, middle and bottom panels). Thus, SPDEF inhibits cell proliferation and decreases Foxm1.

To determine whether SPDEF inhibits cell proliferation, at least in part, through Foxm1, we restored Foxm1 expression in SPDEF OE cells using a lentiviral vector. Increasing the levels of Foxm1 in cultured SPDEF OE cells restored their proliferation to the level of control TRAMP C2 cells (Figure 7A, middle and bottom panels). Flow cytometery demonstrated that increased expression of SPDEF delayed entry of TRAMP C2 cells into S-phase at 12 hours after serum stimulation (Figure 7B, upper and middle panels). Re-expression of Foxm1 in SPDEF OE cells restored the cell cycle progression (Figure 7B, bottom panels) in cultured cells. Furthermore, re-expression of Foxm1 in SPDEF-expressing cancer cells restored tumor weight in orthotopic mouse model of prostate cancer (Figure 7C, left panel), coinciding with increased number of Ki-67-positive (Figure 7D, upper panels) and PH3-positive (Figure 7D, bottom panels) tumor cells. Interestingly, re-expression of Foxm1 in SPDEF-deficient tumor cells restored cell migration (Figure S4A), coinciding with elevated levels of MMP2, MMP9 and MMP13 (Figure S4B). Since SPDEF inhibits cell migration, at least in part, through MMP9 and MM13 [18], increased expression of these MMP genes can contribute to Foxm1-mediated rescue of cell migration in SPDEF-deficient prostate tumor cells. Altogether, these results indicate that SPDEF decreases proliferation and migration of prostate cancer cells through inhibition of Foxm1.

### SPDEF binds to and inhibits the *Foxm1* promoter

**Figure 8:**
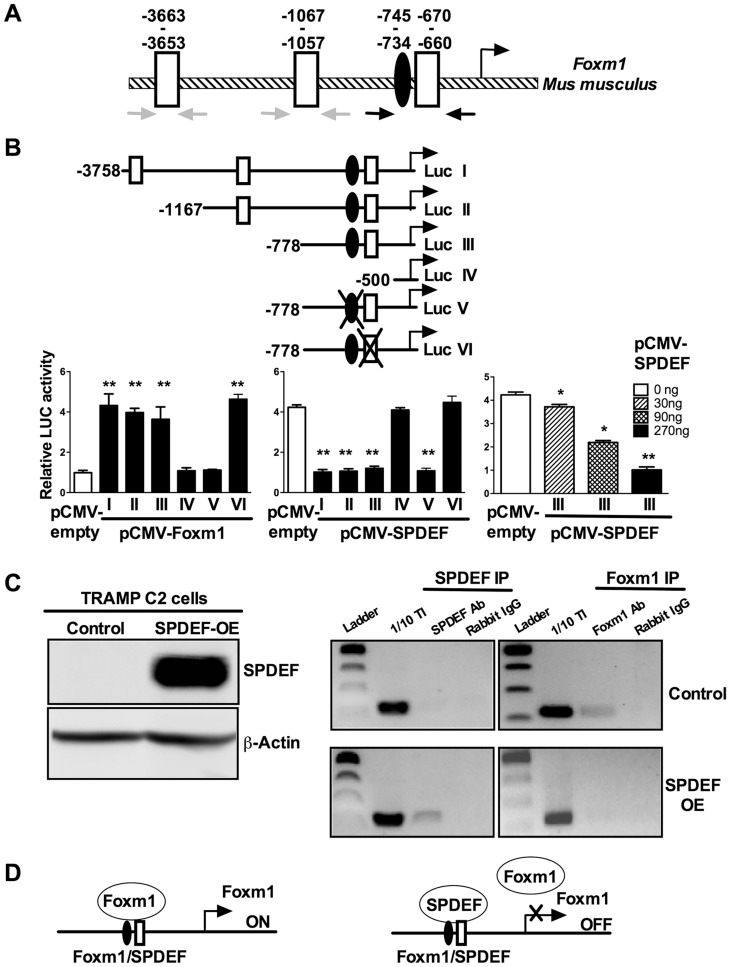
SPDEF represses the Foxm1 promoter. **A**. Schematic drawing of the mouse Foxm1 promoter shows the presence of an evolutionary conserved Foxm1 binding site (black oval) and three SPDEF binding sites (white boxes). **B**. Schematically shown the luciferase (Luc) reporter constructs: Luc I, includes the −3.7 Kb *Foxm1* promoter region; Luc II-IV, include one of its deletion mutants; Luc V, includes a construct with mutations in Foxm1 site; Luc VI, includes a construct with mutations in SPDEF site. TRAMP C2 cells were transfected with CMV-Foxm1b or CMV-SPDEF expression vectors and one of the *Foxm1* promoter LUC plasmids. CMV-empty plasmid was used as a control. Dual LUC assays were used to determine LUC activity. Transcriptional induction is shown as a fold change relative to CMV-empty vector (± SD). A *p* value<0.01 is shown with (**) and *p* value<0.05 is shown with (*). **C**. Western blot shows efficient expression of SPDEF in TRAMP C2 cells after lentiviral transduction (left panel). ChIP assay was performed in control TRAMP C2 cells and TRAMP C2 cells overexpressing SPDEF (SPDEF OE). In control cell, Foxm1 is bound to its own −745/−660 bp promoter region (Foxm1 IP). In SPDEF OE cells, SPDEF is bound to the −745/−660 bp Foxm1 promoter region (SPDEF IP) and the binding of Foxm1 to this region is lost. Neither Foxm1, nor SPDEF bound to the −3663/−3653 and to the −1067/−1057 bp Foxm1 sites (grey arrows in A). **D**. Schematic drawing shows that SPDEF protein physically binds to the −745/−660 bp *Foxm1* promoter region and interferes with Foxm1 binding to the same region.

Since SPDEF expression inversely correlated with Foxm1 expression in mouse and human prostate tumors (Figure 5), we examined the possibility that SPDEF directly represses the Foxm1 promoter. An evolutionary conserved Foxm1 binding site was identified in −745/−734 bp region of the mouse Foxm1 gene. We also identified three potential SPDEF binding sites in the −3.7 Kb Foxm1 promoter region (Figure 8A, schematic drawing). Interestingly, one of them, the −670/−660 SPDEF binding site is located near the Foxm1 binding site, suggesting that SPDEF may influence a positive feedback mechanism regulating the Foxm1 promoter. The −3.7 Kb mouse Foxm1 promoter and its deletion mutants were cloned into luciferase (LUC) reporter vectors and used in co-transfection experiments in TRAMP C2 prostate adenocarcinoma cells (Figure 8B). CMV-Foxm1 plasmid increased activity of the −3.7 Kb Foxm1 promoter (Figure 8B). Deletion of the −778/−500 bp region, containing −745/−734 bp Foxm1 binding site (Luc construct IV), completely abolished Foxm1 promoter activity; whereas deletion of −3758/−1167 bp or −1167/−778 bp regions had no effect (Figure 8B). Thus, the −745/−734 bp Foxm1 site is required for auto-regulation of the −3.7 Kb Foxm1 promoter by Foxm1. Co-transfection with CMV-SPDEF plasmid inhibited *Foxm1* transcriptional activity in a dose-dependent manner (Figure 8B, right panel), indicating that SPDEF is a transcriptional repressor of Foxm1 gene. Interestingly, deletion of the −745/−660 bp SPDEF/Foxm1 site completely abolished the ability of SPDEF to inhibit the *Foxm1* promoter, whereas deletion of −3663/−3653 and −1067/−1057 bp SPDEF sites had no effect (Figure 8B, middle panel). Furthermore, disruption of Foxm1 binding site (construct V, Figure 8B) inhibited Foxm1 promoter activity (Figure 8B and Figure S4C–D), confirming that the −745/−738 bp region is required for the auto-regulatory activation of the Foxm1 promoter. Likewise, disruption of the SPDEF binding site (construct VI, Figure 8B) prevented the inhibitory effect of SPDEF on Foxm1 promoter activity (Figure 8B and Figure S4C), indicating that SPDEF inhibits Foxm1 promoter through the −670/−660 bp region.

We next examined whether SPDEF physically binds to the −745/−660 bp Foxm1 promoter DNA and whether this binding inhibits the ability of Foxm1 to bind to its own binding site. Chromatin Immunoprecipitation (ChIP) assay was performed using TRAMP C2 cells. Since TRAMP C2 cells do not express endogenous SPDEF, we expressed SPDEF by lentiviral infection (Figure 8C, left panel SPDEF OE). In control cells lacking SPDEF, endogenous Foxm1 protein bound to the −745/−660 bp region, whereas there was no binding of SPDEF to this region (Figure 8C, right panels). In SPDEF OE cells, SPDEF bound to the −745/−660 bp Foxm1 promoter region, while the binding of Foxm1 to the same region was lost (Figure 8C, right panels). Neither Foxm1 nor SPDEF bound to the −3663/−3653 and to the −1067/−1057 bp Foxm1 sites. Altogether, SPDEF protein physically binds to the −745/−660 bp *Foxm1* promoter region and directly inhibits the Foxm1 promoter activity by interfering with the ability of Foxm1 to activate its own promoter through an auto-regulatory element in the −745/−660 bp region (Figure 8D).

## Discussion

Previous studies demonstrated that SPDEF is expressed in normal prostate epithelium and prostate tumors in mice and human patients [21], [27]; however, its role in prostate carcinogenesis is still controversial. SPDEF was originally discovered as a transcription factor that directly interacts with androgen receptor and functions as its co-activator to induce expression of prostate specific antigen (PSA) in LNCaP prostate tumor cells [21]. Several groups reported the increased expression of SPDEF during progression of prostate, breast and ovarian cancers, suggesting the oncogenic role of SPDEF [15], [27]. SPDEF was found to be required for tumorigenesis in ER-positive subset of breast cancers [33]. However, the loss of SPDEF during tumor progression was reported by other groups. In advanced prostate cancer and prostate cancer-derived cell lines, SPDEF was either decreased or lost [23], [34]. It was also shown that SPDEF expression is decreased during the transition from low-grade to high-grade prostate cancer [23], [34], [35]. Two studies had shown correlation between decreased expression of SPDEF and poor prognosis in prostate cancer [23], [35]. Likewise, high SPDEF levels were found in prostate cancer patients who had a prolong response to androgen deprivation therapy [36]. Expression and knock-down of SPDEF in different cell lines *in vitro*, provided controversial results [24], [26], [28], [37].

The diverse regulatory factors altering SPDEF expression may explain some of the discrepancies between published studies. Increased SPDEF mRNA, as reported in some studies, might not reflect protein levels of SPDEF, although it was suggested that the protein status of SPDEF strongly correlates with the transcript level [13]. Furthermore, differences in the specificity of antibodies and technical procedures used in each study may have contributed to contradicting results. Alternatively, the prostate cancer cell lines used in the studies have various genetic origins, and this heterogeneity may influence reported findings. While some studies examined SPDEF expression in tumor samples classified by Gleason scores [23], [34], [35], others used pooled tumor samples [27]. Nevertheless, the controversy regarding the role of SPDEF in prostate carcinogenesis remains unresolved. Our present findings provide both *in vivo* and *in vitro* support for tumor suppressive role of SPDEF in prostate cancer. We found inverse correlations between SPDEF and the Foxm1 oncogene in both mouse and human prostate tumors and demonstrated that SPDEF inhibits tumor cells proliferation through Foxm1 oncogene.

Our previous studies demonstrated that aberrant expression of Foxm1 in all cell types using ubiquitous Rosa26 promoter accelerated prostate carcinogenesis in TRAMP and LADY transgenic mice [9]. Prostate cancers contain heterogeneous populations of cells including epithelial, inflammatory and stromal cells that enhances Foxm1 expression during carcinogenesis [38]. Macrophage-specific inactivation of Foxm1 reduced cancer-associated inflammation and decreased tumor growth in chemically-induced lung cancer models [39], indicating that Foxm1 expression in macrophages is important for regulation of cancer-associated inflammation during tumor promotion. Recently, we established that prostate epithelial-specific expression of Foxm1 is required for prostate carcinogenesis. Deletion of Foxm1 from prostate epithelial cells in *PB-Cre/Foxm1^fl/fl^/TRAMP* mice prevented prostate carcinogenesis but did not change SPDEF levels [10]. Critical role of Foxm1 in proliferation of prostate tumor cells was confirmed in orthotopic model using Foxm1-deficient MycCap prostate adenocarcinoma cells [10]. Therefore, understanding the mechanisms regulating Foxm1 is particularly relevant to the pathogenesis of prostate cancer. In the present study, we established that SPDEF directly binds to an evolutionally-conserved region in the Foxm1 promoter and inhibits Foxm1 transcriptional activity via an auto-regulatory element of the Foxm1 promoter. Although, the ability of Foxm1 to activate its own promoter was previously shown, molecular mechanism underlying this regulation was not characterized [40]. Present studies establish, that Foxm1 activates its own promoter by binding to −745/−734 bp site, which is important for auto-regulatory loop. We also identified an evolutionally conserved SPDEF binding site in the close proximity to this Foxm1 binding site and demonstrated that SPDEF prevented Foxm1 binding to its own promoter, inhibits Foxm1 transcriptional activity and decreases expression of Foxm1 targets. Our data demonstrate that SPDEF functions as a tumor suppressor in SV40 T antigens and c-Myc-induced prostate cancers by inhibiting tumor cell proliferation via disruption of an auto-regulatory element in the Foxm1 promoter.

Increased expression of Foxm1 was found in human prostate adenocarcinomas and was correlated with the severity of the disease [8]. At the same time, the decrease in SPDEF expression was associated with transition from low-grade to high-grade human prostate cancer [23], [34], [35]. It is possible that the loss of SPDEF causes increased expression of oncogenic Foxm1, accelerating tumor cell proliferation and leading to poor outcome in prostate cancer patients. Our studies may serve as a foundation for the development of new therapeutic approaches in prostate cancer by targeting Foxm1 via SPDEF dependent pathways.

In summary, decreased expression of SPDEF in prostate epithelial cells was sufficient to increase prostate carcinogenesis, while increased SPDEF inhibited prostate carcinogenesis induced by SV40 T antigens. Decreased prostate carcinogenesis in SPDEF-deficient mice was associated with decreased proliferation of tumor cells and reduced expression of *Cdc25b*, *Cyclin B1*, *Plk-1, AuroraB*, and *Topo2alpha*, factors that are critical for tumor cell proliferation. SPDEF bound to −745/−734 bp Foxm1 promoter region and inhibited the ability of Foxm1 to bind to and activate its own promoter. Our results suggest that the loss of SPDEF during prostate carcinogenesis results in an increased activity of the oncogenic Foxm1.

## Materials and Methods

### Generation of TRAMP-C2R3 and MycCap cell lines expressing SPDEF and Foxm1

SPDEF expression plasmid pLenti-PGK-GFP-SPDEF [41], or Foxm1 expression plasmid pLenti-PGK-GFP-Foxm1 [42], and the control plasmid pLenti-PGK-GFP were used to generate lentiviruses at Cincinnati Children's Hospital Viral Vector Core. TRAMP-C2R3 (termed as TRAMP C2) prostate adenocarcinoma cells [10] and MycCap cells prostate adenocarcinoma cells [43] were transduced with lentiviruses. After two days, GFP expressing cells were sorted using flow cytometry. SPDEF expression was confirmed by qRT-PCR and Western blot.

### Transgenic mice

#### ****Loss-of-function of SPDEF in prostate epithelium****

*SPDEF^−/−^* mice [14] were bred with *TRAMP* transgenic mice containing Pb-driven SV40-T large and t small antigens [6] to generate *SPDEF^−/−^/TRAMP* mice. *SPDEF^−/−^/TRAMP* mice were fertile with no obvious abnormalities. *SPDEF^−/−^ and TRAMP* littermates were used as controls. Mouse prostates were harvested 25 weeks of age.

#### **Gain-of-function of SPDEF in prostate epithelium**

Transgenic mice with doxycycline (Dox)-inducible *Spdef* expression (*TRE-SPDEF*) were generated [13]. The *TRE-SPDEF* mice were bred with *Pb-Cre^tg/+^/LoxP-stop-LoxP-rtTA(Rosa26)^tg/tg^* mice that contained Cre recombinase expressed in prostate epithelial cells under control of rat probasin (PB) promoter and the reverse tetracycline activator (rtTA) inserted into Rosa26 locus [44]. In *Pb-Cre^tg/+^/LoxP-stop-LoxP-rtTA(Rosa26)^tg/+^/TRE-SPDEF* mice (abbreviated as *SPDEF OE*), Dox treatment results in prostate epithelial-specific expression of *SPDEF* transgene due to excision of LoxP-stop-LoxP cassette by Cre recombinase. *SPDEF OE* mice were bred with *TRAMP* transgenic mice to generate *TRAMP/SPDEF OE* mice. To induce *SPDEF*, mice were given Dox in food chow beginning at 4 weeks of age and kept on Dox until the end of the experiment. Dox-treated *TRE-SPDEF/TRAMP* littermates lacking the *Pb-Cre* transgene were used as a control for *Pb-Cre^tg/+^/LoxP-stop-LoxP-rtTA(Rosa26)^tg/+^/TRE-SPDEF*/*TRAMP* transgenic mice. Additional controls included *Pb-Cre^tg/−^/TRE-SPDEF ^tg/−^* or *Pb-Cre^tg/+^/LoxP-stop-LoxP-rtTA(Rosa26)^tg/+^/TRE-SPDEF*/*TRAMP* mice without Dox.

#### Orthotopic model of prostate cancer

5×10^5^ TRAMP C2R3 cells, expressing exogenous SPDEF, Foxm1 or GFP, were injected into the prostates of anesthetized C57BL/6 mice. Mouse prostates were harvested 4 weeks after the surgery. All animal studies were approved by the Animal Care and Use Committee of Cincinnati Children's Hospital.

### Immunohistochemistry

The following antibodies were used for immunochistochemistry: anti-Foxm1 [42], [45], anti-SPDEF [46], anti-Ki67 (Thermo Scientific) and anti-phospho-Histone H3 (pH 3) antibody (Santa Cruz).

### Cell growth assay

Control TRAMP C2 and SPDEF OE TRAMP C2 cells, or control MycCap and SPDEF OE MycCap cells were plated in triplicate. Alive cells were counted at 1, 2, 3, and 4 days using hemocytometer or WST-1 reagent (Roche) according to the manufacturers' recommendations. Optical density was quantified at 450 nm using spectrophotometer. Standard curves were made with increasing numbers of each of the prostate cancer cell lines to convert fluorescent readings into cell numbers. Experiments were performed in triplicates and presented as average numbers of cells ± S.D.

### *In vitro* scratch wound healing assay

Control TRAMP C2 cells or SPDEF OE TRAMP C2 were plated onto 6-well plates and allowed to grow into confluent monolayers. Scrape wounds were generated using a 20- µl pipette tip, and cell media were replaced. Phase-contrast images of the cells were taken at 0 h, 18 h and 24 h after wounding, and the average wound closure rate was measured. The width of the scratch was quantified using ImageJ (National Institutes of Health). The relative migration distance was calculated by dividing the width of scratch at each time point by the width of the scratch at time zero. The relative distance was then converted to a percentage by multiplying by 100. Experiments were done in triplicate.

### Soft agar assay

For soft agar assay, control and SPDEF OE TRAMP C2 cells were plated on soft agar for two weeks to assay for anchorage-independent cell growth. The culture medium containing 10% fetal calf serum was replaced every 3 days. Colonies containing more than 50 cells were scored after 2 weeks. Triplicate plates were used to count colonies and determine the mean number of colonies ± SD.

### Quantitative real-time RT-PCR (qRT-PCR)

Total RNA was prepared from mouse prostates and analyzed by qRT-PCR using the StepOnePlus Real-Time PCR system (Applied Biosystems, Foster City, CA) as described [46]. RNA was amplified with Taqman Gene Expression Master Mix (Applied Biosystems) combined with inventoried Taqman mouse gene expression assays: *Foxm1*, Mm00514924_m1; *β-Actin*, Mm00607939_g1; *Cdc25b*, Mm00499136_m1; *Cyclin B1*, Mm00838401_g1; *Plk1*, Mm00440924_g1; *SPDEF*, Mm01306245_m1; *Topo2α*, Mm00495703_m1; *Cyclin A2*, Mm00438063_m1; *Aurora B*, Mm01718146_g1; *Cks1b*, Mm01617993_gh. Reactions were analyzed in triplicates and expression levels were normalized to *β-actin* mRNA.

### Analysis of SPDEF in human prostate cancer

Data from human prostate cancer microarray datasets, GSE21034 [30] and GSE16560 [31] were downloaded from the GEO archive [47]. For the GSE21034 dataset, expression measurements were first log2-tranformed and expression levels of SPDEF and FOXM1 genes were compared between metastatic and primary tumor samples. Three different probesets representing three different FOXM1 transcripts were available for the FOXM1 gene. Expression of each transcript was analyzed separately. Statistical analysis of differences in median expression levels for transcripts between the two sample groups were performed using the non-parametric Wilcoxon rank test. For the GSE16560, samples were split in two different ways. First, expression levels were compared between indolent and lethal prostate cancers. Second, high-risk and low-risk sample groups were formed as described in [32]. High-risk samples were derived from patients surviving less than 12 months and low-risk samples from patients surviving more than 192 months. Statistical analysis was again performed using the non-parametric Wilcoxon rank test. Survival analysis was performed by stratifying patients in GSE16560 dataset [31] by the expression level of FOXM1 and SPDEF genes into “low” (lowest third), “middle” (middle third) and “high” (highest third) expression groups. Statistical analysis of Kaplan-Meier survival curves for different strata was performed using the log-rank test [48]. All microarray data analysis was performed using R and Bioconductor packages [49]. In all comparisons, p-values less than or equal to 0.05 were considered to be statistically significant.

### Cloning the mouse *Foxm1* promoter region and luciferase assays

Mouse genomic DNA was used to amplify the −3758 bp to +1 bp region of the mouse *Foxm1* promoter (Gene Bank Number NC_000072.6) using the primers 5′-GGT ACC TTT CTG GGA CTG TCT GCG-3′ and 5′-GAG CTC AGC GCC GCT TTC AGT TG-3′. To create deletion mutants of the −3758 bp *Foxm1* promoter region, we used the following primers: −1167, 5′-GGT ACC GTG CTG GAA TTA AAG GTG TGC-3′ and 5′-GAG CTC AGC GCC GCT TTC AGT TG-3′; −778, 5′-GGT ACC GAA CGG CTT TAC TGT CCT AAG-3′ and 5′-GAG CTC AGC GCC GCT TTC AGT TG-3′; −500, 5′-GGT ACC CTT ATC GTA AAG TAC TTC GAG GG-3′ and 5′-GAG CTC AGC GCC GCT TTC AGT TG-3′. PCR products were cloned into a pGL3 firefly luciferase (LUC) reporter plasmid (Promega, Madison, WI) and verified by DNA sequencing. Site-directed mutagenesis was used to mutate several nucleotides in either Foxm1 or SPDEF site of the −778 bp Foxm1 promoter LUC construct (Suppl. Fig. 4D). *FoxM1B*-LUC constructs were co-transfected with CMV-*FoxM1B* [50], CMV-SPDEF or CMV-empty plasmids [41] in TRAMP-C2R3 cells using Lipofectamine (Invitrogen). CMV-*Renilla* was used as an internal control to normalize the transfection efficiency. A dual luciferase assay (Promega) was performed 24 hours post transfection as described previously [51].

### ChIP assay

Control and SPDEF-overexpressing TRAMP-C2R3 cells were cross-linked by addition of formaldehyde, sonicated and used for immunoprecipitation with rabbit anti-SPDEF antibody (H-250, Santa Cruz) or rabbit anti-Foxm1 antibody [10] as described previously [52]. Rabbit polyclonal IgG (Vector Lab) was used as ChIP negative controls. DNA fragments were between 500 bp and 1000 bp in size. Reversed cross-linked ChIP DNA samples were subjected to PCR amplification with oligonucleotides specific to promoter regions of mouse Foxm1: −3758/−3551 (5′-TTT CTG GGA CTG TCT GCG-3′ and 5′-CCT TGT TAG CCT GAT GTC ATG-3′); −1169/−966 (5′-GTG CTG GAA TTA AAG GTG TGC-3′and 5′-AGG GTC TTC GCC TTT CTG-3′); −778/−562 (5′-GAA CGG CTT TAC TGT CCT AAG-3′and 5′-GAG GGA GCA CAG AAT GAG-3′).

### Cell cycle analysis

Control and SPDEF expressing TRAMP-C2R3 cells were serum starved for 36 hr by using DMEM media supplemented with 0.1% FBS. A complete media with 10% FBS was added and cells were collected at the indicated time points, fixed in 70% ethanol, and stained with propidium iodide/RNase solution (Cell Signaling). Flow cytometry was used to count cells in G0/G1, S and G2/M phases of cell cycle.

### Statistical analysis

Microsoft Excel was used to calculate SD and statistically significant differences between samples using the Student T-Test. P values<0.05 were considered statistically significant.

## Supporting Information

Figure S1 SPDEF^−/−^ mice did not develop prostate tumors in the absence of TRAMP transgene and cellular proliferation in SPDEF^−/−^ prostates was unchanged. **A**. Ki67 staining of SPDEF^−/−^ and control wild type prostates shows the lack of aberrant proliferation in prostate tissues. Slides were counterstained with nuclear fast red. **B**. qRT-PCR shows the lack of *SPDEF* mRNA in SPDEF^−/−^ prostates. mRNAs of *Foxm1*, *Cdc25b*, *cyclin B1* and *cyclin D1* were unchanged. mRNA levels were normalized to *β-actin* mRNA. A *p* value<0.05 is shown with (*).
(TIF)

Figure S2 Lentiviral expression of SPDEF in MycCap prostate adenocarcinoma cells decreased cell growth *in vitro*. **A**. Transgenic expression of SPDEF in TRAMP C2 was compared to human prostate adenocarcinoma cell lines using qRT-PCR. **B**. *SPDEF* mRNA is increased in SPDEF OE cells (left panel) shown by qRT-PCR. Transgenic expression of SPDEF in TRAMP C2 was compared to human prostate adenocarcinoma cell lines using qRT-PCR (right panel). **C**. Overexpression of SPDEF in MycCap cells reduced mRNAs of cell cycle regulatory genes. *β-actin* mRNA was used for normalization. **D**. Overexpression of SPDEF decreased proliferation of MycCap adenocarcinoma cells *in vitro*. Control and SPDEF-expressed MycCap cells were seeded in triplicates and counted at different time points using WST1 Cell Proliferation Reagent (left panel) or hemocytometer (right panel). A *p* value<0.05 is shown with (*).
(TIF)

Figure S3 *SPDEF* expression was inversely correlated with *Foxm1* expression in human prostate tumors. The raw data for human prostate cancer microarray dataset GSE21034 was used. SPDEF and FOXM1 mRNAs were compared between Metastatic and Primary tumor samples. Three different probe sets representing three different FOXM1 transcripts were available for the FOXM1 gene.
(TIF)

Figure S4 SPDEF inhibits tumor cell migration through transcriptional repression of Foxm1 gene. **A**. Re-expression of Foxm1 in the SPDEF-positive prostate adenocarcinoma cells restored tumor cell migration *in vitro*. Wound healing assay was used to measure cell migration. **B**. Expression levels of migration-specific genes were analyzed using qRT-PCR. *β-actin* mRNA was used for normalization. **C**. Schematic drawings of promoter regions of the mouse Foxm1 gene is shown on the left. Locations of the Foxm1 binding site and SPDEF binding site are indicated by the oval and square shape (WT-Luc). Site-directed mutagenesis was used to disrupt either Foxm1 site (Foxm1 mut-Luc) or SPDEF site (SPDEF mut-Luc). Mutated nucleotides are indicated with red letters. The mutated luciferase plasmids and CMV plasmids expressing Foxm1 or SPDEF were used to co-transfect TRAMP C2 cells. Luc was measured to determine promoter activity (right panels). Transcriptional induction is shown as a fold change relative to CMV-empty vector (±SD) and a p value<0.01 is shown with (**). **D**. Evolutionary conserved binding sites in the Foxm1 promoter. Basic Local Alignment Search Tool (BLAST) was used to align Foxm1 promoter sequences from mouse, rat and human. In addition to a 50-bp strictly conserved sequence at the transcription start site, conserved Foxm1 and SPDEF binding sites were found in the promoter.
(TIF)
