# Supplementary figures and images for: SPDEF Inhibits Prostate Carcinogenesis by Disrupting a Positive Feedback Loop in Regulation of the Foxm1 Oncogene
Source: PLoS Genet. 2014 Sep 25;10(9):e1004656. doi: 10.1371/journal.pgen.1004656 (PMC4177813; doi:10.1371/journal.pgen.1004656)

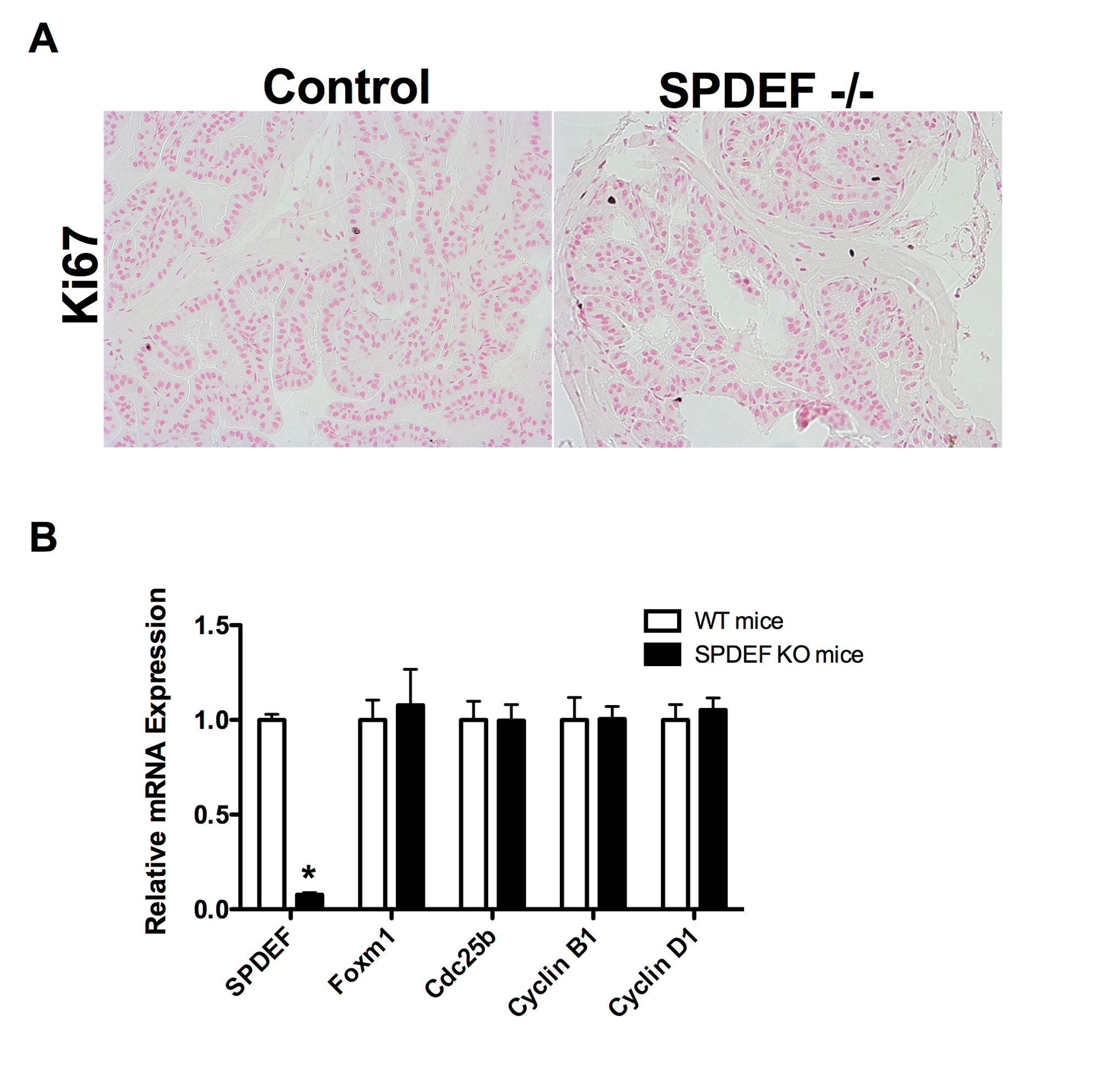

Supplement: Figure S1 — SPDEF−/− mice did not develop prostate tumors in the absence of TRAMP transgene and cellular proliferation in SPDEF−/− prostates was unchanged. A. Ki67 staining of SPDEF−/− and control wild type prostates shows the lack of aberrant proliferation in prostate tissues. Slides were counterstained with nuclear fast red. B. qRT-PCR shows the lack of SPDEF mRNA in SPDEF−/− prostates. mRNAs of Foxm1, Cdc25b, cyclin B1 and cyclin D1 were unchanged. mRNA levels were normalized to β-actin mRNA. A p value<0.05 is shown with (*). (TIF) [file pgen.1004656.s001.tif]

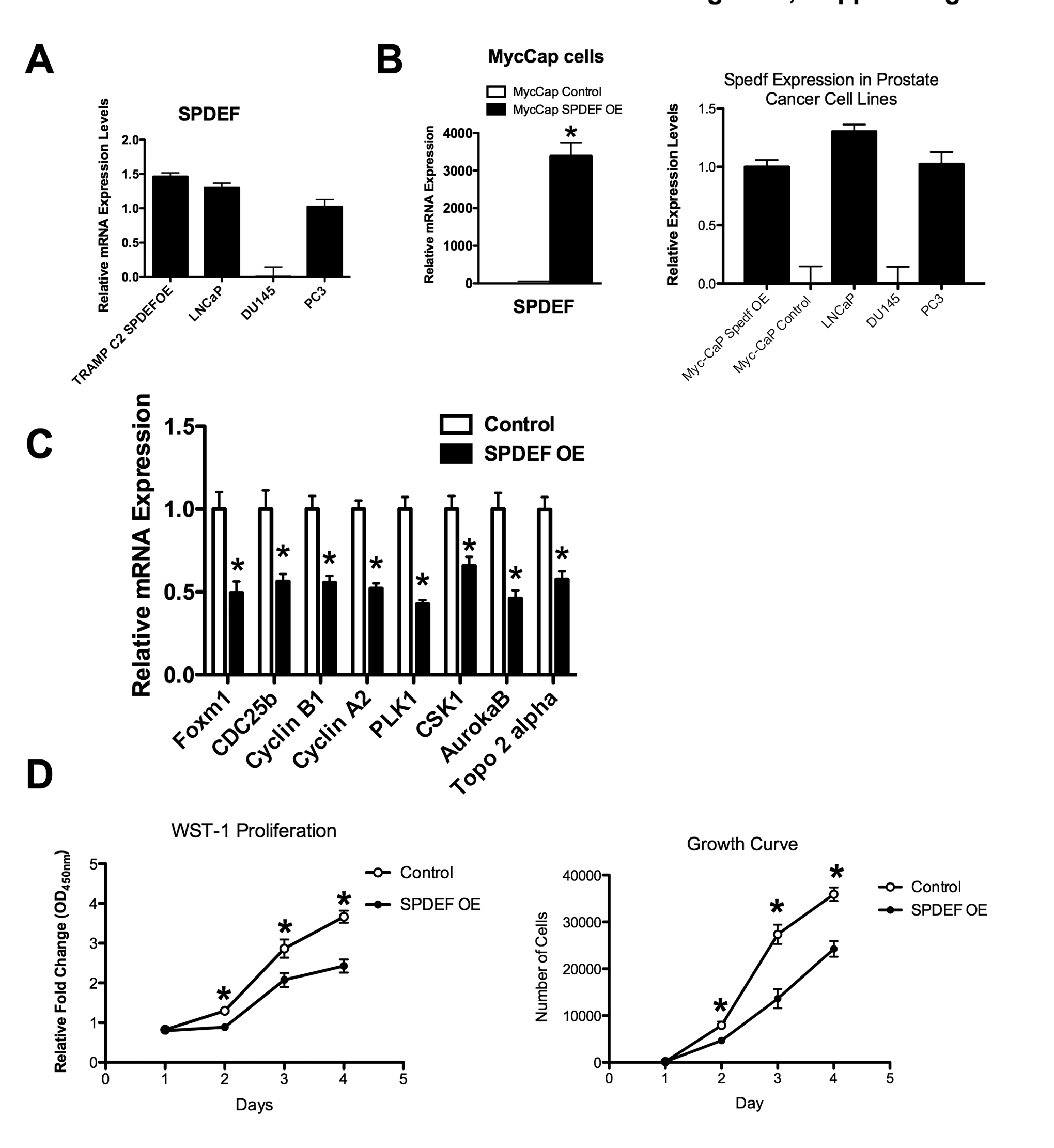

Supplement: Figure S2 — Lentiviral expression of SPDEF in MycCap prostate adenocarcinoma cells decreased cell growth in vitro. A. Transgenic expression of SPDEF in TRAMP C2 was compared to human prostate adenocarcinoma cell lines using qRT-PCR. B. SPDEF mRNA is increased in SPDEF OE cells (left panel) shown by qRT-PCR. Transgenic expression of SPDEF in TRAMP C2 was compared to human prostate adenocarcinoma cell lines using qRT-PCR (right panel). C. Overexpression of SPDEF in MycCap cells reduced mRNAs of cell cycle regulatory genes. β-actin mRNA was used for normalization. D. Overexpression of SPDEF decreased proliferation of MycCap adenocarcinoma cells in vitro. Control and SPDEF-expressed MycCap cells were seeded in triplicates and counted at different time points using WST1 Cell Proliferation Reagent (left panel) or hemocytometer (right panel). A p value<0.05 is shown with (*). (TIF) [file pgen.1004656.s002.tif]

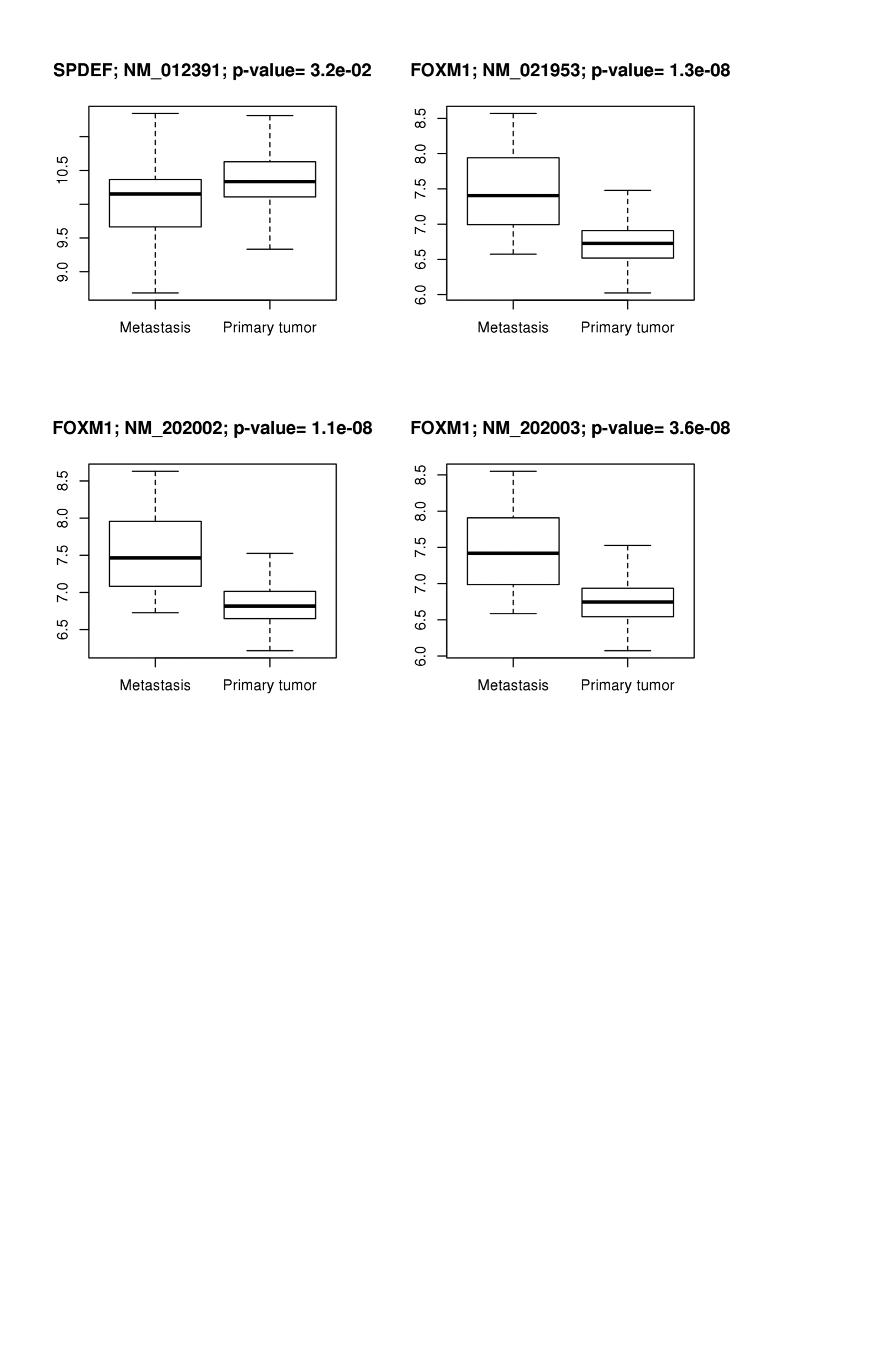

Supplement: Figure S3 — SPDEF expression was inversely correlated with Foxm1 expression in human prostate tumors. The raw data for human prostate cancer microarray dataset GSE21034 was used. SPDEF and FOXM1 mRNAs were compared between Metastatic and Primary tumor samples. Three different probe sets representing three different FOXM1 transcripts were available for the FOXM1 gene. (TIF) [file pgen.1004656.s003.tif]

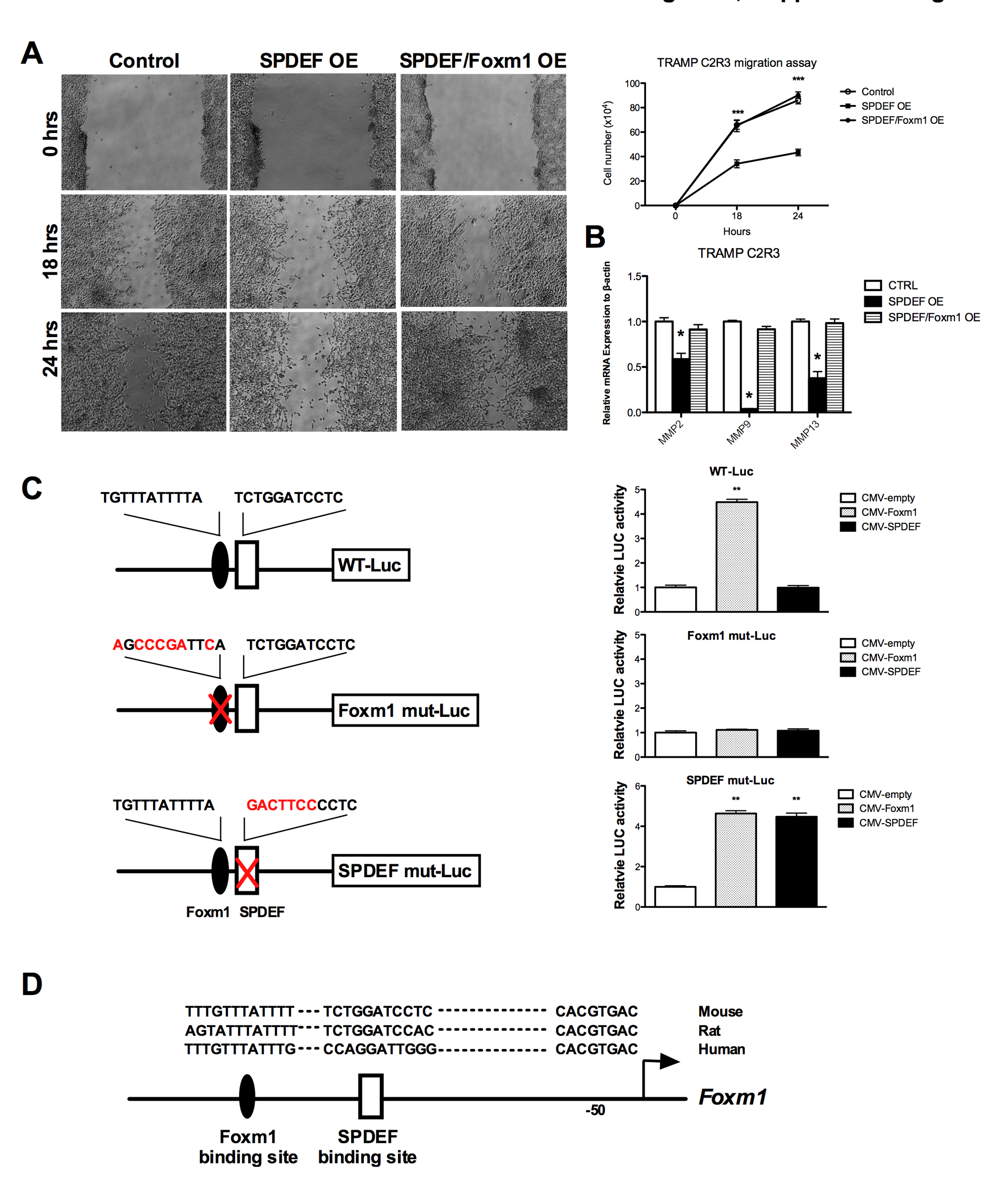

Supplement: Figure S4 — SPDEF inhibits tumor cell migration through transcriptional repression of Foxm1 gene. A. Re-expression of Foxm1 in the SPDEF-positive prostate adenocarcinoma cells restored tumor cell migration in vitro. Wound healing assay was used to measure cell migration. B. Expression levels of migration-specific genes were analyzed using qRT-PCR. β-actin mRNA was used for normalization. C. Schematic drawings of promoter regions of the mouse Foxm1 gene is shown on the left. Locations of the Foxm1 binding site and SPDEF binding site are indicated by the oval and square shape (WT-Luc). Site-directed mutagenesis was used to disrupt either Foxm1 site (Foxm1 mut-Luc) or SPDEF site (SPDEF mut-Luc). Mutated nucleotides are indicated with red letters. The mutated luciferase plasmids and CMV plasmids expressing Foxm1 or SPDEF were used to co-transfect TRAMP C2 cells. Luc was measured to determine promoter activity (right panels). Transcriptional induction is shown as a fold change relative to CMV-empty vector (±SD) and a p value<0.01 is shown with (**). D. Evolutionary conserved binding sites in the Foxm1 promoter. Basic Local Alignment Search Tool (BLAST) was used to align Foxm1 promoter sequences from mouse, rat and human. In addition to a 50-bp strictly conserved sequence at the transcription start site, conserved Foxm1 and SPDEF binding sites were found in the promoter. (TIF) [file pgen.1004656.s004.tif]
